# Personalized medicine—a tradition in general practice!

**DOI:** 10.1080/13814788.2019.1589806

**Published:** 2019-04-29

**Authors:** Manfred Maier

**Affiliations:** Department of General Practice, Centre for Public Health, Medical University of Vienna, Vienna, Austria


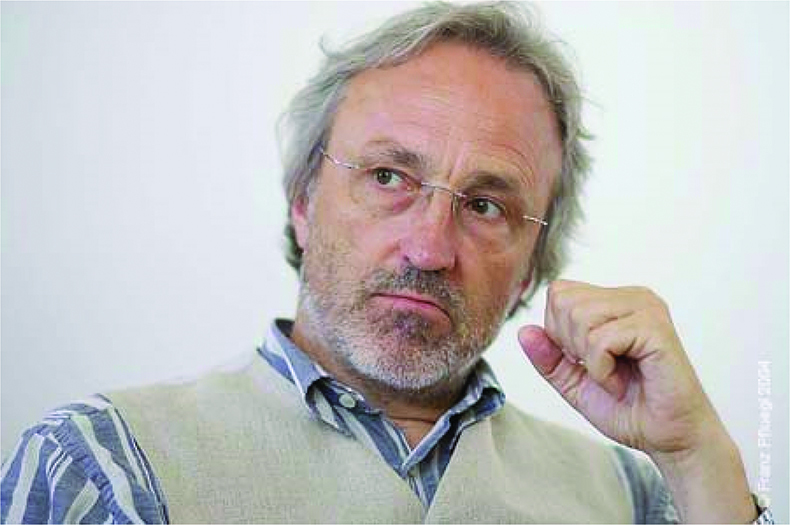
Nowadays, ‘personalized medicine’—often used interchangeably with ‘precision medicine’—is a trendy topic in popular and medical media. The term suggests a significant new development in clinical medicine; authors often claim a paradigm shift in healthcare. University hospitals proudly announce the founding or planning of initiatives dedicated to research in ‘precision medicine’ and politicians are eager to provide the funds for these important medical developments [1].

With our emphasis on tailored care for the individual patient, evidence being one ingredient for decision-making but personal preferences and circumstances of the patient playing a major role as well, one might wonder whether general practitioners (GPs) have not always been practicing ‘personalized’ medicine.

## What is ‘personalized medicine’?

Let us first have a look at some definitions. The Centre for Disease Control explains: **‘**Precision medicine, sometimes called personalized medicine, is an approach for protecting health and treating disease that takes into account a person’s genes, behaviours, and environment. Interventions are tailored to individuals or groups, rather than using a one-size-fits-all approach in which everyone receives the same care’ [2]. The National Research Council explains: ‘Precision Medicine refers to the tailoring of medical treatment to the individual characteristics of each patient. It means the ability to classify individuals into subpopulations that differ in their susceptibility to a particular disease, in the biology or prognosis of those diseases they may develop, or in their response to a specific treatment. Preventive or therapeutic interventions can then be concentrated on those who will benefit, sparing expense and side effects for those who will not’ [3]. The medical specialty most often referred to in relation to ‘precision medicine’ is oncology. General applications are the identification of individual risk factors for developing a disease (disease susceptibility), prediction of the response to pharmacological treatment (pharmacogenetics) or determination of the likelihood for serious adverse events after a drug is given.

These definitions and explanations should immediately remind us about a similar term, describing one of the core competencies of our discipline: the ‘person-centred approach.’ As the Wonca definition reads: ‘The general practitioner/family doctor develops a person-centred approach, orientated to the individual, his/her family, and their community. Family medicine deals with people and their problems in the context of their life circumstances, not with impersonal pathology or “cases”. It is as important to understand how the patients cope with and view their illness as dealing with the disease process itself. The common denominator is the person with his or her beliefs, fears, expectations and needs’ [4].

From comparing these various definitions, it becomes clear that the terms ‘personalized medicine/precision medicine’ and ‘person-centred medicine’ are in fact all referring to the most important focus of high-quality care: the person in front of the doctor asking for advice or help. To provide optimal medical care, the experienced GP always had to take into account the detailed history of individual patients and their families (‘genetics’), their individual background in terms of social and occupational circumstances (‘environment’) and their individual approach to health and disease such as adherence to therapy, coping mechanism and lifestyle (‘behaviour’). Each experienced GP also knows the challenge of treating patients with the same condition but with a different response to identical treatments, e.g., for pain, hypercholesterolaemia, hypertension or diabetes. As a result, in general practice, treatment of individual patients routinely is ‘personalized’ or ‘individualized.’

## Personalized medicine is nothing new

Obviously, the traditional approach of GPs to individual peoplès health problems is quite similar to the meaning of ‘personalized or precision medicine’ and is, therefore, nothing new. Just remember the famous quotes by William Osler from the nineteenth century: ‘It is more important to know what sort of person has a disease than to know what sort of disease a person has.’ His advice to students was ‘Care more particularly for the individual patient than for the special features of the disease’ [5]. Only the methods accessible to practice personalised medicine are nowadays different and new: examples are the tests for BRCA1 or BRCA2 mutation to determine the risk of breast and ovarian cancer or the test for CYP2C9 polymorphism to predict the optimal dosage of coumarin derivatives before treatment is initiated. While high-tech specialist medicine increasingly relies on genetic tests or ‘-omics’ technologies, GPs pursue the same goals by low cost methodology: continuously learning about the family history of their patients, which is relevant to build a sound knowledge over time about individual genetic risks, behaviour, response to treatment or adherence [6].

## What is new?

Despite the similarities between the ‘person-centred approach’ in general practice and ‘personalized or precision medicine’ in some specialities, there is, in fact, a major paradigm shift in medicine and in healthcare in general. For the management of patients in terms of diagnostic and therapeutic procedures, GPs and specialists alike have become accustomed in the past to consider disease-specific guidelines with the aim of standardizing the care for all patients with a given condition. Healthcare administrators and policy-makers saw standardization in healthcare as a way to improve patient care and at the same time keep healthcare affordable. On the other hand, practitioners are critical about the concept of using practice guidelines as ‘cookbook medicine’ or as the only ‘correct recipe’ for patient care. As Wonca World’s former president Richard Roberts once said: ‘Guidelines are for standard patients … but I have never met a standard patient!’ (Wonca Europe Conference, Basel, 2009). Therefore, the scientific developments in genetics and in genetic testing to identify subtypes of various diseases or subtypes of responses to drug treatment have now paved the way to the broader recognition of tailoring medical treatment to the specific characteristics of individual patients with a disease. This shift away from thoughtlessly using a ‘one-size-fits-all’ approach to medical care can be considered as progress and relief. Moreover, is such an approach not evidence-based medicine at a state-of-the-art level?

## Could new developments in personalized medicine be helpful in primary care?

Since the achievements of the Human Genome Project, genetic tests are increasingly available and their high costs will eventually get lower over time. At the higher levels of healthcare and at the direct consumer level their use for individual patients likely will increase. For primary care, it will undoubtedly take more time until relevant genetic tests for point-of-care use will be accessible. If affordable for public healthcare systems, such a development would certainly help to inform decisions of the family doctor. However, collecting comprehensive information about the individual family history of patients (‘primary care genetics’) and using this knowledge for their management will certainly remain to be a pillar of high-quality general practice at low cost for the healthcare system [7].

In conclusion, the concepts of ‘personalized medicine’ and ‘precision medicine’ as well as ‘person-centred medicine’ refer to the ability to use hereditary information to ‘personalize’ treatment. This approach has existed in medicine since time immemorial, in particular in the field of general practice. However, recent technical advances in genetic testing allow assessing this information of individual patients more accurately.

Manfred Maier *Department of General Practice, Centre for Public Health, Medical University of Vienna, Vienna, Austria*
manfred.maier@meduniwien.ac.at

